# Large scale enterohemorrhagic *E coli* population genomic analysis using whole genome typing reveals recombination clusters and potential drug target

**DOI:** 10.12688/f1000research.17620.3

**Published:** 2020-09-01

**Authors:** DJ Darwin Bandoy

**Affiliations:** 1 Department of Veterinary Paraclinical Sciences, University of the Philippines Los Baños, Los Baños, Laguna, 4031, Philippines

**Keywords:** pangenome, pharmacophore, EHEC, Escherichia coli

## Abstract

Enterohemorrhagic *Escherichia coli *continues to be a significant public health risk. With the onset of next generation sequencing, whole genome sequences require a new paradigm of analysis relevant for epidemiology and drug discovery. A large-scale bacterial population genomic analysis was applied to 702 isolates of serotypes associated with EHEC resulting in five pangenome clusters. Serotype incongruence with pangenome types suggests recombination clusters. Core genome analysis was performed to determine the population wide distribution of sdiA as potential drug target. Protein modelling revealed nonsynonymous variants are notably absent in the ligand binding site for quorum sensing, indicating that population wide conservation of the sdiA ligand site can be targeted for potential prophylactic purposes. Applying pathotype-wide pangenomics as a guide for determining evolution of pharmacophore sites is a potential approach in drug discovery.

## Introduction

One of the more prominent strains of *Escherichia coli* is the enterohemorrhagic *E. coli* (EHEC) pathotype associated with global outbreaks of bloody diarrhea and hemolytic uremic syndrome (HUS) usually by consumption of undercooked beef ^1^. Within the cattle reservoir, sdiA gene is required by *E. coli* to survive within the acidic rumen environment. SdiA is used by *E. coli* to sense acyl homoserine in a quorum sensing system ^2^. However, it is considered as an orphan as the cognate acyl homoserine synthase is absent, and hence sdiA is considered an environmental sensor to sense the nearby microbial community. SdiA is stabilized by acyl homoserine lactone and acts as transcription factor glutamate decarboxylase needed for survival in the acidic environment. Hence blocking the ability of EHEC to survive the acidic ruminal environment is a proposed mechanism to control shedding in the cattle reservoir.

Whole genome sequencing of bacterial pathogens, particularly EHEC, is quickly transforming the workflows of epidemiological investigations. However, most bioinformatic pipelines used in clinical investigation perform data reduction of genomes and artificially reduce diversity due to comparison of a limited number of housekeeping genes ^3^. While wgMLST attempts to increase the number of genes for analysis, the assignment of a single reference genome appears to be inadequate in light of the pangenome. Various studies have shown that a significant number of genes that are present to the entire universe of genes within a species are missed for variant calling if only a single reference gene is used ^4^. In this study, a multi-scale approach was applied to generate genome wide clustering using the entire pangenome, composed of the core genome and the accessory genome via variable k-mers ^5^. This approach allows differentiation between clusters as well as within serotypes, which is a limitation of using low resolution techniques like MLST.

The concept of the pangenome, which represents the entirety of the genes that are present within a species, which can also be adjusted to the pathotype level, was applied in this particular study. The EHEC pangenome represents the combination of genes seen in the EHEC pathotype. While a prior pangenome of *E. coli* contained 17 genomes, I generated and updated EHEC pangenome with 702 genomes, representing the largest population wide whole genome comparison to date ^6^. The pangenome enables clustering of isolates using gene presence and absence. Targetting the core genome, represented in this study by sdiA, enables integration of population genomics with drug discovery target identification. This strategy enables to capture the pangenome wide variation and ensures all conserved variants are targeted by the drug discovery pipeline coupling the pangenome to pharmacophore modelling.

## Methods

### EHEC population

EHEC associated serotypes are defined based on a previous study ^7^. This study defined EHEC strains as subgroup of Shiga-toxin producing *E. coli* and are belonging to the following serotypes (O26:H11,O45:H2,O103:H2,O111:H8,O121: H19, O145:H28, and O157:H7). Whole genome sequences with the associated EHEC metadata was downloaded from Enterobase 1.1.2 using the keyword search of the respective serotypes within the *E. coli* species ^8^. This search yielded 702 genomes from environmental, animal and clinical samples. (Underlying data: Metadata from Enterobase 1.1.2 of EHEC pangenome ^9^). As this genomes are different from version 1 of this paper, previous Figure 1 was deleted and new Figure 1A was generated reflecting the expanded genomes used in the analysis.

### EHEC pangenome

Whole genome typing in the context of the pangenome was performed using PopPUNK (POPulation Partitioning Using Nucleotide Kmers) 1.1.6. ^5^. The genomes were annotated with Prokka 1.13.3 as per published protocol ^10^. Gff files were extracted as input for the pangenome pipeline Roary 3.11.2 using the following parameters for not splitting paralogs (roary -s -p 32 *.gff) and the resulting presence absence matrix together with the accessory genome phylogeny visualized in Phandango 1.3.0 and is represented as Figure 1B ^11^. Each blue bar represents an individual gene and solid blue blocks represent gene clusters. Previous Figure 1B was deleted and new version of Figure 1B was regenerated integrating the new genomes.

**Figure 1A.**
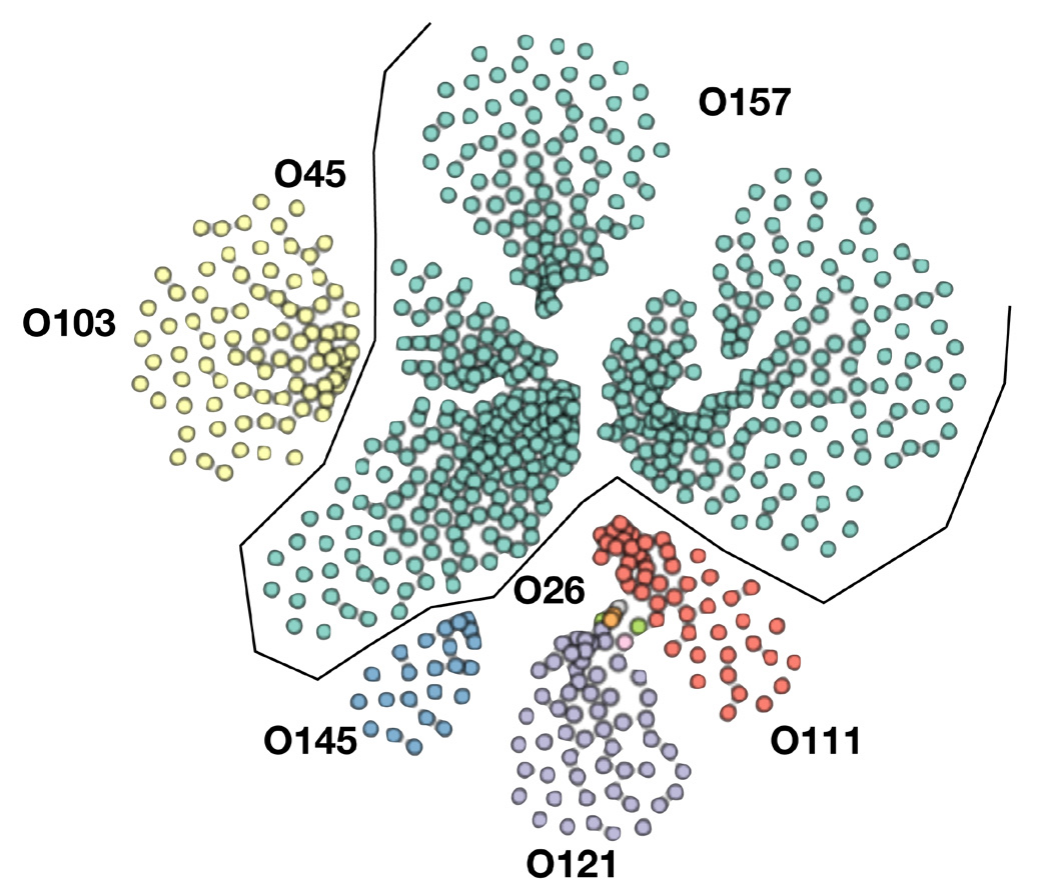
Pangenome wide clustering using k-mers. There are three clusters within the 0157 serotype, 026 is clustered with O111 as well as 103 with O45. Previous Figure 1A was replaced to reflect the increase in genomes analyzed.

**Figure 1B.**
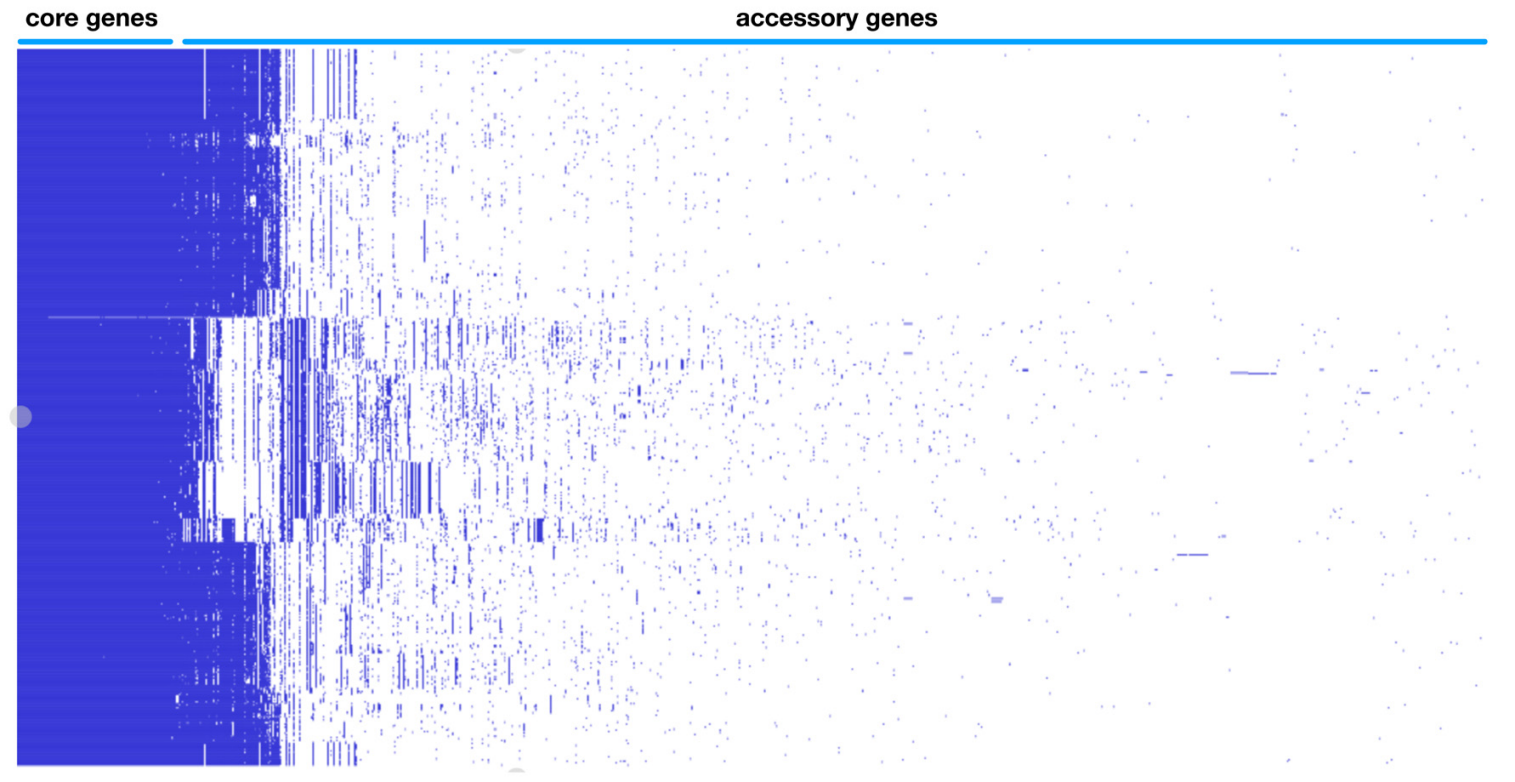
EHEC pangenome showing genomic diveristy with the gene presence absence variation matrix. Previous Figure 1B was replaced to reflect the increase in genomes analyzed.

### Allelic variant calling

Snippy variant calling pipeline 4.3.5 was used to determine the synonymous and nonsynonymous protein mutations using sdiA of *Escherichia coli* O157:H7 str. Sakai as reference. The –contigs option was added to the standard commandline (snippy –outdir –ref sdiA_sakai.gbk). The resulting individual variants of sdiA was merged into EHEC *E. coli* sdiA variant calling data (Underlying data ^9^). Previous Figure 3 in version was removed as the new data was better represented by a new Table 2. McDonald-Kreitman test was done using the Snippy output containing data on synonymous and nonsynonymous mutations ^12^.

### *In silico* sdiA protein modelling

SdiA genes were extracted from the pangenome output of Roary and protein *in silico* modelling performed using SWISS-MODEL ^13– 17^. SdiA protein sequences were used as targets to search for protein templates within the SWISS-MODEL library. Model selection was based on the template with the highest quality prediction by the target-template alignment.

## Results and discussion

Pangenome based clustering integrated the core and accessory elements was applied on 702 whole genomes sequences from serotypes associated with EHEC from diverse sources in the environment as well as animal and human hosts capture the evolutionary space. The majority of the available sequences are from O157 H7 representing 68.5% (481 out of 702) and the rest from the other major non-O157 serotype designated as the “big six”, with O45 H2 1.9% (13 out of 702), O103 H2 10.7% (77 out of 702), O26 H11 1.3% (9 out of 702), O111 H8 6.0% (42 out of 702), O121 H19 8.1% (57 out of 702) and O145 H28 3.2% (23 out of 702). The variable-length k-mer analysis and comparison software (PopPUNK) enables scalable, annotation and alignment free approach to large scale population genomics ^5^. The accessory genome details the recent acquisition of mobile elements via horizontal gene transfer conveying metabolic, virulence and antibiotic resistance properties which cannot be captured by classical approaches. Eliminating an integral property of recombigenic organism underestimates the diversity and artificially creates similarity and relatedness. The analysis yielded five major pangenomic clusters of EHEC associated isolates. Cluster I is represented by O157 with three genomic subclusters, cluster two contains serotypes O103 and O45, cluster III contains serotype O121, cluster IV contains serotypes O26 and O111 and cluster V contains serotype O145 ( Figure 1A). This updated analysis expanded the genomes from version 1 of this paper with 152 genomes into 702 which necessitates the regeneration of Figure 1. A better visualization of the pangenome cluster was also utilized. Clusters containing several serotypes like cluster II and IV indicate that recombination events blur the genomic boundary resulting to being meshed together in a gradient of dots visually. This novel genome wide framework allows a greater resolution of comparison, as it is now possible to compare similar organisms within the same serotype and determine specific lineages integrating the accessory genome. The acquisition of genomic islands unique to individual isolates are well defined in the pangenome gene presence absence matrix ( Figure 1B). The core genome is 2966 ( Table 1) and total gene count within the EHEC pangenome is 27774, exceeding previous estimates of total *E. coli* pangenome 22,000. This enormous difference between the core gene and total gene highlights the variation between the different isolates, which can be strain specific and individual isolate specific as indicated by the pangenome data. However, further analysis is limited due to the incompleteness of the metadata entry with regards to the pertinent parameters such specific geolocation, organ of isolation, severity of clinical signs and others.

**Table 1.** Pangenome metrics.

	Percentage Occurence	
Core genes	(99% <= strains <= 100%)	2966
Soft core genes	(95% <= strains < 99%)	301
Shell genes	(15% <= strains < 95%)	2889
Cloud genes	(0% <= strains < 15%)	21618
Total genes	(0% <= strains <= 100%)	27774

SdiA is a core gene found across the EHEC pangenome clusters based on the genome wide pangenome analysis, indicating that it can be a suitable interventional target. Considering the huge diversity between pangenome clusters, sdiA homology was analyzed and compared. Remarkably, pangenome cluster I showed highly conserved sdiA structure across global spatial and temporal range (30 years), in spite of cluster I diverging to three separate subsclusters. Divergence from the canonical sdiA structure is more prominent in other genomic clusters. Pangenome cluster II yielded the most number of nonsynonymous mutations (50%) in sdiA gene ( Table 2). The percentage distribution for the rest of the pangenome clusters are as follows: 22% for cluster IV, 21% for cluster III and 4% for cluster V. The topological relevance of the predominant mutations was further contextualized by protein modelling.

**Table 2.** Nonsynonymous mutations summary integrating the pangenome clusters.

EHEC Pangenome Cluster	Serotype	Nonsynonymous mutation position		
		101_240	140_240	189_240
II	O103H2	77	77	77
IV	O111H8	40	40	40
III	O121H19	55	55	
V	O145H28	23		
I	O157H7	2	2	1
II	O45H2	13	13	13
	Total	210	187	131

The impact of the most prevalent nonsynonymous mutations were analyzed with protein modelling using sdiA of *Escherichia coli* O157:H7 str. Sakai as template. The most ranked nonsynonymous mutation is asparagine to serine at amino acid position 101 with 39.1% (210/536 located adjacent to η-4 phenylalanine which is associated with the ligand docking ( Figure 2B). This is followed by 24.4% (131/536) of the nonsynonymous mutation is due to conversion of arginine to lysine at position 189 of sdiA ( Figure 2A). This amino acid is located with the α-6 domain, adjacent to the amino acid clusters associated with sdiA dimerization. Previous protein modelling determined the role of guanidinium group of arginine which enables interactions in three different directions enabling a more complex electrostatic interaction versus lysine as well as the higher pKa value in arginine that can yield a more stable ionic interaction compared to lysine ^18^. β-5 domain alanine to threonine change at amino acid position 140 is the third ranked nonsynonymous mutation with 34.9% (187/536) ( Figure 2C). None of the highly ranked nonsynonymous mutations impact the ligand interaction, indicating the conservation of the sdiA motif across the population in geographic and temporal distribution, which suggests the possibility of targeting sdiA for quorum sensing inhibition. Mutational analysis using McDonald-Kreitman test indicate differential selection pressures between serotypes. Serotypes O103:H2,O45:H2 and O111:H8 have slightly higher between group nonsynonymous/synonymous ratios (0.42,0.45,0.43 respectively) than within species nonsynonymous/synonymous ratios (0.375 using O157:H7 as within species group). Serotypes O145:H28, O121:H19, O26:H11 have lower values compared to the within species values (0.33, 0.22,0 respectively).

**Figure 2A.**
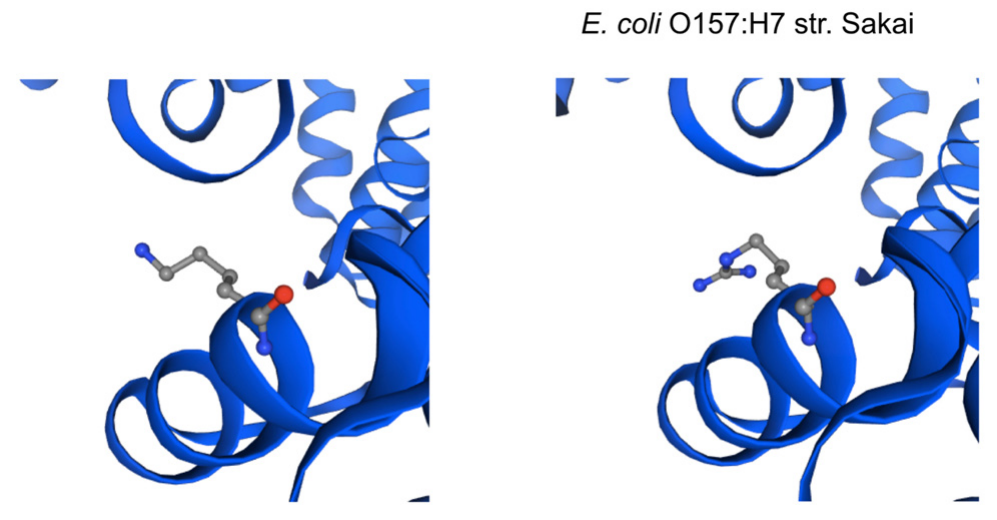
Protein model of the nonsynonymous variant at amino acid position 189.

**Figure 2B.**
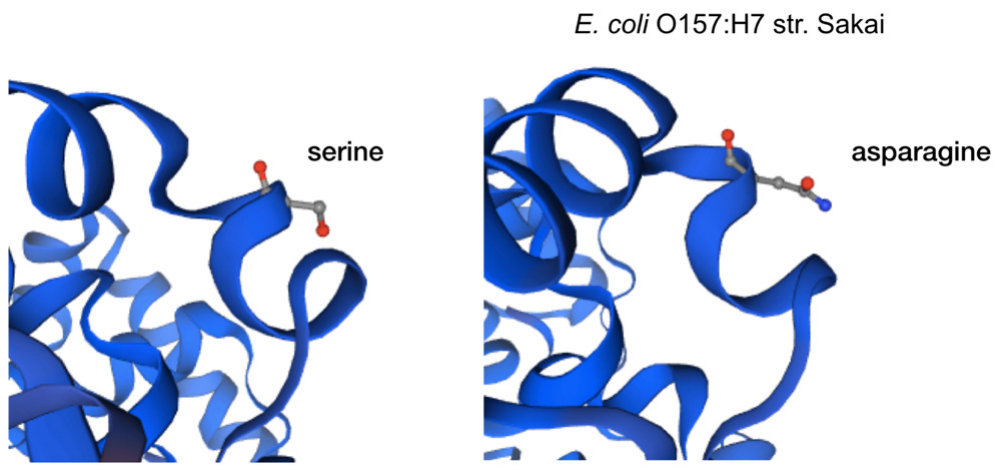
Protein model of the nonsynonymous variant at amino acid position 101.

**Figure 2C.**
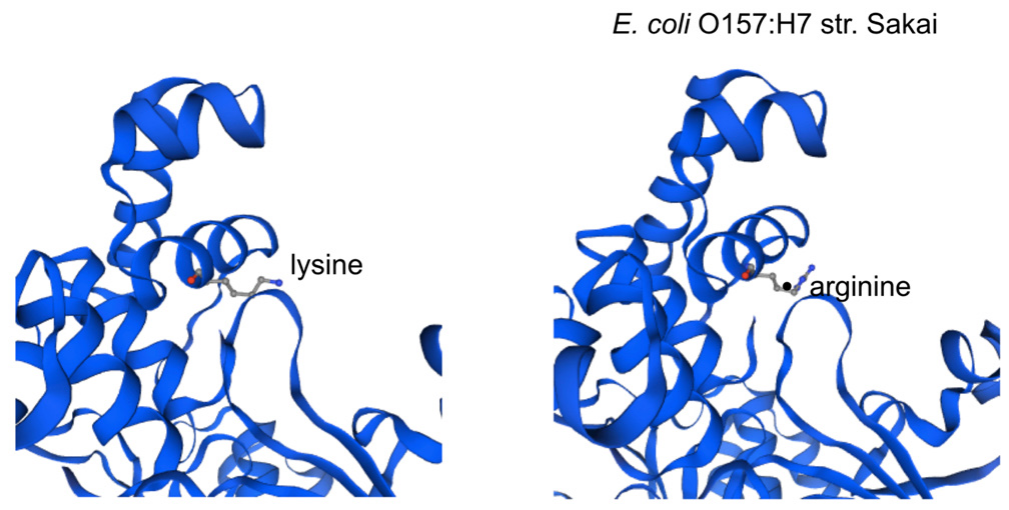
Protein model of the nonsynonymous variant at amino acid position 140.

## Conclusion

While EHEC pangenome is remarkably diverse, the allelic variants of sdiA, particularly nonsynonymous mutants, indicate the conservation of quorum sensing domain, indicating that targeting this structure can be effective across the different lineages of EHEC pathotype.

## Data availability

All underlying and extended data available from Open Science Framework: Supplemental Data for Pangenome guided pharmacophore modelling of enterohemorrhagic *Escherichia coli* sdiA, https://doi.org/10.17605/OSF.IO/BNZ85 ^9^

Data are available under the terms of the Creative Commons Zero "No rights reserved" data waiver (CC0 1.0 Public domain dedication).

### Underlying data

Table 1 Metadata from Patric Database of EHEC *E. coli* pangenome, version 1 replaced with the updated 702 genomes

Table 2 EHEC *E. coli* pangenome presence absence matrix, version 1 replaced with the updated 702 genomes

Table 3 EHEC *E. coli* sdiA variant calling data, version 1 replaced with the updated 702 genomes

### Extended data

SWISS-MODEL Homology Modelling Report available at osf.io/bnz85.
