# Supplementary material for: Large scale enterohemorrhagic E coli population genomic analysis using whole genome typing reveals recombination clusters and potential drug target
Source: F1000Res. 2020 Sep 1;8:33. Originally published 2019 Jan 9. [Version 3] doi: 10.12688/f1000research.17620.3 (PMC7610179; doi:10.12688/f1000research.17620.3)
Supplement: Peer review history

# Peer review: Reviewer response for version 3

**DOI:** 10.5256/f1000research.29079.r70557

**Published:** 2020-11-03

**Authors:** Olivier Tenaillon

**Affiliations:** 1 IAME (Infection Antimicrobials Modelling Evolution), UMR 1137, French Institute of Health and Medical Research (INSERM), Paris, France

Table 2 is not properly labelled. Positions should be presented without the _240 and eventually the precise mutation named. It should be mentionned that the numbers refers to number of strains carrying the mutation.

 Results from the MK test indicate low conservation. Which outgoup has been used? If the outgroup is too close too few mutations will be present to have reliable value. More details should be given on how the tests have been preformed

 Apart from these minor comments the manuscript is fine.

Reviewer Expertise:

NA

I confirm that I have read this submission and believe that I have an appropriate level of expertise to confirm that it is of an acceptable scientific standard.

# Peer review: Reviewer response for version 2

**DOI:** 10.5256/f1000research.22447.r54557

**Published:** 2019-11-06

**Authors:** Kerry K. Cooper

**Affiliations:** 1 School of Animal and Comparative Biomedical Sciences, University of Arizona, Tucson, AZ, USA

While I disagree with the author that "The inclusion of non-EHEC is necessary as outgroup comparison groups and does not debunk the validity of the analysis pipeline". As including non-EHEC genomes in an EHEC core genome analysis does alter the results of the output EHEC core genome, which is evident by the author removing those genomes from the analysis.

 However, the author has done an excellent job of incorporating a huge amount of only EHEC serotype genomes into the analysis, and as a result has generated a much stronger study. The second version of this manuscript is tremendously better, and the author's additional work has made it significantly higher quality paper. 

 The only comments are extremely minor:

- In the results and discussion section: Amino acid position 140 is ranked third with 34.9%, but the position 189 is 2nd with 24.4% frequency, these should be switched. 
- I would also recommend switching the Figure 2A,B, and C around, so that they are introduced in order of A, B and C.

Otherwise, no further comments.

Reviewer Expertise:

I am an expert in foodborne bacterial genomics, epidemiology and pathogenesis, particularly  E. coli, Salmonella,  Campylobacter, and Listeria.

I confirm that I have read this submission and believe that I have an appropriate level of expertise to confirm that it is of an acceptable scientific standard.

# Peer review: Reviewer response for version 2

**DOI:** 10.5256/f1000research.22447.r54556

**Published:** 2019-10-29

**Authors:** Olivier Tenaillon

**Affiliations:** 1 IAME (Infection Antimicrobials Modelling Evolution), UMR 1137, French Institute of Health and Medical Research (INSERM), Paris, France

I think this version is better than the previous one, but I still think the connection to sidA could be made stronger and the relevance of the focus on that gene and this subgroup also. Any conserved gene could be a target for drug. So is this gene more interesting than others in that group, the question should be more precise from the beginning.

 The focus on some nonsynymous mutations is interesting, but if these mutations are neutral there is not much interest in showing a detailed structure. A precise Ka/Ks study should be done to tell if these few non synonymous are more than expected.

Reviewer Expertise:

NA

I confirm that I have read this submission and believe that I have an appropriate level of expertise to confirm that it is of an acceptable scientific standard, however I have significant reservations, as outlined above.

# Peer review: Reviewer response for version 1

**DOI:** 10.5256/f1000research.19267.r46991

**Published:** 2019-05-15

**Authors:** Olivier Tenaillon

**Affiliations:** 1 IAME (Infection Antimicrobials Modelling Evolution), UMR 1137, French Institute of Health and Medical Research (INSERM), Paris, France

The present manuscript presents a state-of-the-art pan genome analysis of EHEC strains and a subsequent analysis of the variation in sdiA.

 The analysis of sdiA could have been completed with simple KA/Ks analysis and compared to that of the core genome. For now, there is no connection between the two analysis, the authors could have simply performed the analysis of sdiA.

 Some mutants have frame shifts in the gene, this is not discussed.

 I think the author could try to connect more the two parts of the analysis.

Is the work clearly and accurately presented and does it cite the current literature?

Yes

If applicable, is the statistical analysis and its interpretation appropriate?

Yes

Are all the source data underlying the results available to ensure full reproducibility?

Yes

Is the study design appropriate and is the work technically sound?

Partly

Are the conclusions drawn adequately supported by the results?

Partly

Are sufficient details of methods and analysis provided to allow replication by others?

Yes

Reviewer Expertise:

Microbial genomics

I confirm that I have read this submission and believe that I have an appropriate level of expertise to confirm that it is of an acceptable scientific standard, however I have significant reservations, as outlined above.

# Response

**Published:** 2019-05-15

**Authors:** DJ Darwin Bandoy

**Affiliations:** University of the Philippines, Philippines

I thank the reviewer for the effort in doing the review. I accept all the suggestions and will add the population genetic analysis in the next version of the paper.

# Peer review: Reviewer response for version 1

**DOI:** 10.5256/f1000research.19267.r47169

**Published:** 2019-04-24

**Authors:** Kerry K. Cooper

**Affiliations:** 1 School of Animal and Comparative Biomedical Sciences, University of Arizona, Tucson, AZ, USA

I would state the biggest issue with the manuscript is the work is not technically sound, because upon examining the metadata file from Patric, numerous strains included in the EHEC pangenome were in fact not EHEC strains. Many of the isolates are UPEC, STEC or EPEC strains that should not be included in the analysis and are resulting in potential errors in the results and conclusions of the manuscript. All O55 strains are EPECs, O104 from the Germany outbreak is a STEC not EHEC, O127 is an EPEC, and CFT073 is an UPEC strain, just as some examples.

 Furthermore, the analysis also includes O157:H7 strains that are wild type and mutant strains, and the mutants should not be included in the analysis. Additionally, the authors mention three clades include O157 (Clade I), O26 (Clade II) and O80 (Clade III), however these clades are formed because the vast majority of the strains used in the analysis come from those three serotypes. The analysis is missing three serotypes from the "big 6" serotypes, including O45, O121 and O145, and only include one or two representatives of two of the other serotypes O111, O103. There are numerous genomes available for each of these serotypes through NCBI that should be included in this analysis. Particularly as the "big 6" serotypes represent >50% of the infections, and in the United States represent adulterants in ground beef or other meat products. Therefore, they are a vital aspect for the development of pharmacophore modelling of *sdiA* to prevent colonization in cattle.

 Additionally, several genomic studies by Ogura et al (2009) ^1^ and Cooper et al (2014) ^2^ have shown that many of these "big 6" serotypes arise along different evolutionary pathways or split from O157 at different time points thus acquire different genes. It would vital to include these in analysis to see if these different pathways impacted the conservation of *sdiA*. The author should also provide a much cleaner version of the metadata as a separate tab in the spreadsheet that includes only those strains that were included in the analysis. Unfortunately, the above-mentioned issue means that all of the results in the manuscript are potential erroneous and need to be completely re-done with the elimination of non-EHEC strains and the inclusion of additional "big 6" genomes to provide a scientifically sound analysis.

 It would also be helpful to include in the methods section the date of the search, as the database is constantly changing making reproduction a little bit easier by other researchers. Upon the new analysis it would be helpful to include a brief table or statement of the serotype breakdown included in the EHEC pangenome that would also eliminate some of the above-mentioned issues and make it easier for readers to get a sense of those serotypes included in the analysis. 

 There are a number of grammatical errors or poor phrasing in the manuscript that should be reviewed and corrected. Such as there is only one author and no indication of other researchers on the manuscript, yet the manuscript keeps stating we instead of I. 

 Finally, there are several points made in the introduction and discussion that do not have references. For example, in the introduction the author mentions "pangenome of *E. coli *was published in 2008 contained 8 genomes" but does not reference the paper. Additionally, the author mentions "serogroup O80 has aside from Shiga toxin, an extra-intestinal virulence plasmid (pS88), is currently emerging in France" but do not reference anything indicating the emergence in France. I have also provided the citations for Ogura et al. ^1^ and Cooper et al. ^2^ for the author to review and potentially cite in the manuscript.

Is the work clearly and accurately presented and does it cite the current literature?

Partly

If applicable, is the statistical analysis and its interpretation appropriate?

Not applicable

Are all the source data underlying the results available to ensure full reproducibility?

Yes

Is the study design appropriate and is the work technically sound?

No

Are the conclusions drawn adequately supported by the results?

Partly

Are sufficient details of methods and analysis provided to allow replication by others?

Yes

Reviewer Expertise:

I am an expert in foodborne bacterial genomics, epidemiology and pathogenesis, particularly  E. coli, Salmonella,  Campylobacter, and Listeria.

I confirm that I have read this submission and believe that I have an appropriate level of expertise to state that I do not consider it to be of an acceptable scientific standard, for reasons outlined above.

## References

[1] : Comparative genomics reveal the mechanism of the parallel evolution of O157 and non-O157 enterohemorrhagic Escherichia coli. *Proc Natl Acad Sci U S A*.2009;106(42) : 10.1073/pnas.0903585106 17939-44 10.1073/pnas.0903585106 19815525PMC2764950

[2] : Comparative genomics of enterohemorrhagic Escherichia coli O145:H28 demonstrates a common evolutionary lineage with Escherichia coli O157:H7. *BMC Genomics*.2014;15: 10.1186/1471-2164-15-17 17 10.1186/1471-2164-15-17 24410921PMC3893438

# Response

**Published:** 2019-05-15

**Authors:** DJ Darwin Bandoy

**Affiliations:** University of the Philippines, Philippines

I appreciate the work of the reviewer for going through the metadata. The inclusion of non-EHEC is necessary as outgroup comparison group and does not debunk the validity of the analysis pipeline. In light of this clarification, since this is the sole reason for the non-approval, I appeal for an approval with reservations as the analysis is technically and scientifically sound. 

I am in the process of including the big 6 serotypes in the analysis based on the reviewer's comments, as well as the additional references which are very constructive additions to the paper. Again, as the reviewer sees the value in redoing a more inclusive analysis of EHEC serotypes, this is another justification to approve with reservation the paper submitted.

I beg to disagree with the comment of using "inclusive we" in place of I as a grammatical error. The use of royal or inclusive we in lieu of I is a matter of preference. This is the only part of the review I do not agree with.
